# Movement Behaviour of the Carabid Beetle *Pterostichus melanarius* in Crops and at a Habitat Interface Explains Patterns of Population Redistribution in the Field

**DOI:** 10.1371/journal.pone.0115751

**Published:** 2014-12-31

**Authors:** Bas Allema, Wopke van der Werf, Joop C. van Lenteren, Lia Hemerik, Walter A. H. Rossing

**Affiliations:** 1 Farming Systems Ecology, Wageningen University, Wageningen, The Netherlands; 2 Laboratory of Entomology, Wageningen University, Wageningen, The Netherlands; 3 Crop & Weed Ecology Group, Centre for Crop Systems Analysis, Wageningen University, Wageningen, The Netherlands; 4 Biometris, Wageningen University, Wageningen, The Netherlands; Centro de Investigacion Cientifica y Educacion Superior de Ensenada, Mexico

## Abstract

Animals may respond to habitat quality and habitat edges and these responses may affect their distribution between habitats. We studied the movement behaviour of a ground-dwelling generalist predator, the carabid beetle *Pterostichus melanarius* (Illiger). We performed a mark-recapture experiment in two adjacent habitats; a large plot with oilseed radish (*Raphanus sativus*) and a plot with rye (*Secale cereale*). We used model selection to identify a minimal model representing the mark-recapture data, and determine whether habitat-specific motility and boundary behaviour affected population redistribution. We determined movement characteristics of *P. melanarius* in laboratory arenas with the same plant species using video recording. Both the field and arena results showed preference behaviour of *P. melanarius* at the habitat interface. In the field, significantly more beetles moved from rye to oilseed radish than from radish to rye. In the arena, habitat entry was more frequent into oilseed radish than into rye. In the field, movement was best described by a Fokker-Planck diffusion model that contained preference behaviour at the interface and did not account for habitat specific motility. Likewise, motility calculated from movement data using the Patlak model was not different between habitats in the arena studies. Motility (m^2^ d^−1^) calculated from behavioural data resulted in estimates that were similar to those determined in the field. Thus individual behaviour explained population redistribution in the field qualitatively as well as quantitatively. The findings provide a basis for evaluating movement within and across habitats in complex agricultural landscapes with multiple habitats and habitat interfaces.

## Introduction

Conservation biological control requires the presence of natural enemies at the right time and place. Understanding how movement of natural enemies contributes to patterns of their high or low densities is therefore of great importance. One framework to study natural enemy movement is that of spillover [1]. Tscharntke and colleagues [2] posed this framework as one of eight hypotheses on the role of landscape composition and configuration in determining the structure of ecological communities, ecosystem functioning and services. One aspect that needs to be elucidated within this framework is the difference in species' edge responses, especially which types of edges maximise spillover and how habitat size, configuration, quality and edge effects are related ([2] and references therein). While the ecological responses to habitat edges are well documented [3], [4] detailed discussion on edge behaviour is only now emerging [5]. This discussion will benefit from detailed studies on movement behaviour in combination with a study on the population outcome of this behaviour.

Animals may respond to habitat edges in various ways. They might for example readily traverse the edge in one direction but not in the other. This behaviour can cause an accumulation of individuals at one side of the edge. For instance, Haynes and Cronin [6] found that the plant hopper *Prokelisia crocea* traversed an edge between its host patch and a hostile matrix primarily in one direction (matrix to host patch), causing accumulation in the patch. Asymmetry in behaviour at an edge can cause a gradient in density [7]. Another possible edge behaviour is that movement is inhibited at both sides of the edge, e.g. when a road, ditch or canal is separating two habitats. Another possibility is that individuals have a strong drive to leave a habitat irrespective of the side they are coming from. Analytical solutions to edge behaviour of animals within the diffusion framework can be found in Cantrell and Cosner [8], Ovaskainen and Cornell [9] and Maciel and Lutscher [10]. While carabids are a cornerstone in biological control of crop pests, there is little information on their behaviour at habitat edges. The only experimental study on edge behaviour with a carabid beetle that we are aware of was conducted by Bommarco and Fagan [11]. They found that inclusion of edge behaviour in a model for population redistribution improved predictions. However, they could not separately identify how movement within habitats and across the habitat interface affects the population pattern.

When we know the behaviour of an animal in different habitat types and on the edges between them predictions can be made on the population spread at landscape level [10], [12]. Ecologists who have incorporated detailed and realistic behaviour into the movement process have accomplished better fits to movement data [13]. Quantifying individual variation in movement or behavioural parameters has great potential for linking individual movements to population re-distribution [14]. We try to make this link by looking on the one side to the individual behaviour and the other side to population redistribution.

We studied individual behaviour and population redistribution in the carabid beetle *Pterostichus melanarius* (Illiger) in two different crops and near the edge between them. *Pterostichus melanarius* is a common carabid species in agricultural land and is considered an important natural enemy for the biological control of several pest species [15], [16], [17] while it can also affect other natural enemies [18]. *Pterostichus melanarius* emerges from pupae in June [19] and spillover of this abundant predator across habitat edges may have a significant impact on trophic relationships in neighbouring habitats.

First, we present a field mark-recapture experiment on the carabid beetle *Pterostichus melanarius* in two adjacent habitats consisting of oilseed radish (*Raphanus sativus*) on the one hand and rye (*Secale cereale*) on the other hand. A model is fitted to these mark-recapture data to address the question how habitat-specific motility and boundary behaviour contribute to population redistribution in an agricultural landscape context. Secondly, we present empirical data on movement behaviour of *P. melanarius* in laboratory arenas planted with the same two plant species to link individual behaviour to population redistribution. Model parameters are calculated from the movement data to compare findings at the individual level (bottom-up) with findings at the population level (top-down).

## Methods

### 2.1 Field experiment

Dispersal of adult females of *P. melanarius* was measured in 2009 in an area of 229×52 m at the organic farm Droevendaal, Wageningen, the Netherlands (51°59′N, 5°39′E). This farm is part of the research facilities of Wageningen University, and no specific permission was required for conducting the field work. Oilseed radish (*Raphanus sativus*; var. Brutus) was grown on half of the field, and rye (*Secale cereale;* var. Admiraal) on the other half. Both crops were sown in the first week of August 2009. An imaginary line between the adjacent rows of oilseed radish and rye was taken to be the habitat interface. The field was surrounded by a 3–6 m wide grass margin, which on the north side included 1.5–2.5 m tall shrubs and trees.

Female adult beetles for release in the field experiments were collected from a grass/clover field in the weeks preceding the experiment using pitfall traps. Beetles were stored in containers (45×30×15 cm; about 200 beetles per container) on a substrate of moist potting soil in a dark room at 4°C and fed frozen fly maggots (*Lucilia caesar*). A few days before release, the beetles were marked with a dot of nail polish (OPI Nail lacquer NL B777/H41) on the elytra. Different colours were used to distinguish beetles released in radish from those released in rye.

**Figure 1:**
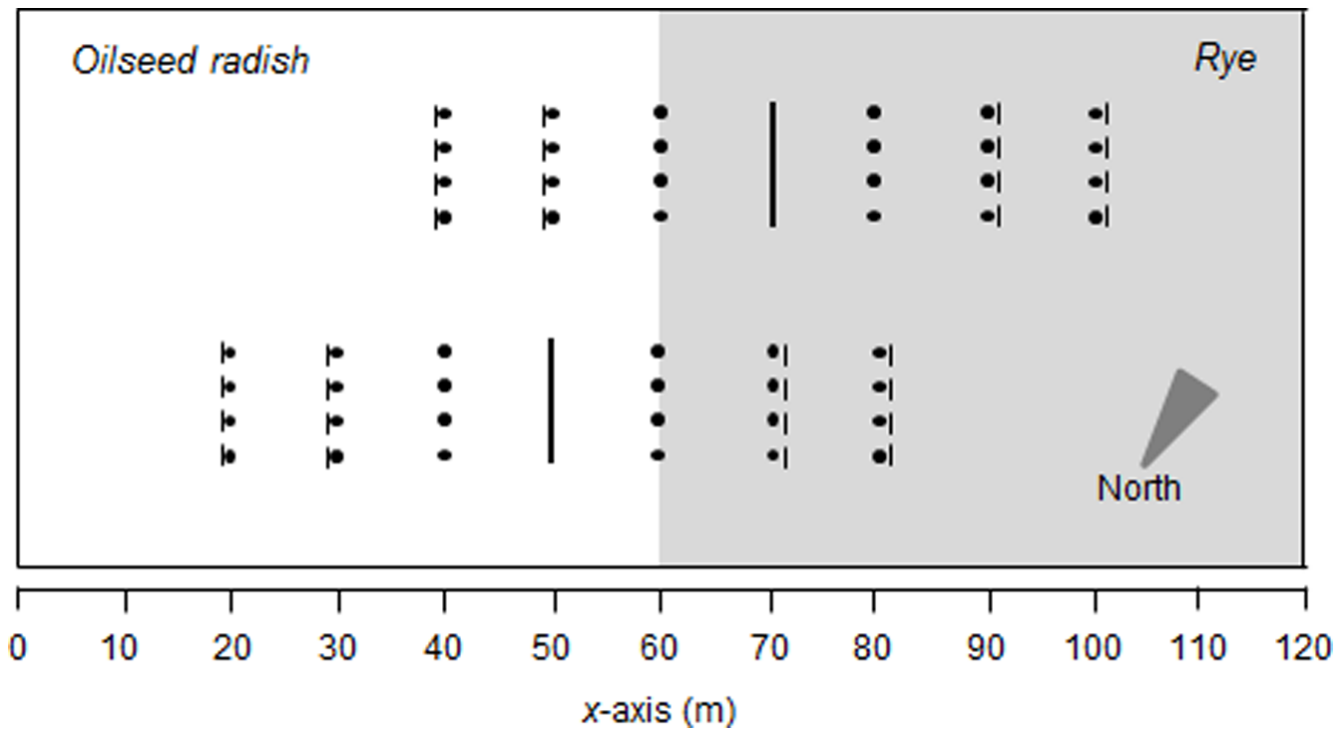
Spatial layout of the field experiment at Droevendaal organic experimental farm in Wageningen, the Netherlands. *Pterostichus melanarius* were released at the long vertical lines and recaptured at trapping stations with (|•, •|) or without (•) screens.

Releases were made at 6 PM on 7 September 2009. Release points were organized in a line at a constant distance (10 m) from the crop interface (Fig. 1). In both oilseed radish and rye, 1015 beetles were released. Recaptures were made using 8.5 cm diameter pitfall traps placed in lines at 10, 20, and 30 m at either side of the release lines. There were eight pitfalls per distance, and they were organized in four sets of two pitfalls, with 50 cm between the pitfalls within the same set, and 140 cm between sets. Pitfall traps at 20 and 30 m from the release line were equipped with screens to enhance trapping, while traps at 10 m from the release line did not have screens to minimize interference with dispersal (Fig. 1). Traps were sampled 17 times over a period of 23 days, until 30 September 2009. Recaptured beetles were removed from the experiment. Results were pooled per trapping station.

#### 2.1.1 Analysis of mark-recapture data

We defined 16 alternative versions of a Fokker-Planck diffusion model with or without preferential movement at the habitat interface to simulate carabid dispersal. Each of the alternative models was fitted to data using maximum likelihood, and Akaikes information criterion was used to weigh goodness of fit (negative log likelihood) against the number of parameters and select the model(s) most supported by the data [20], [21]. Models contained terms accounting for (1) random movement, (2) interface-mediated behaviour, (3) loss of beetles due to trapping, and (4) loss of beetles due to mortality and mark wear. A basic model without edge behaviour would be described as:
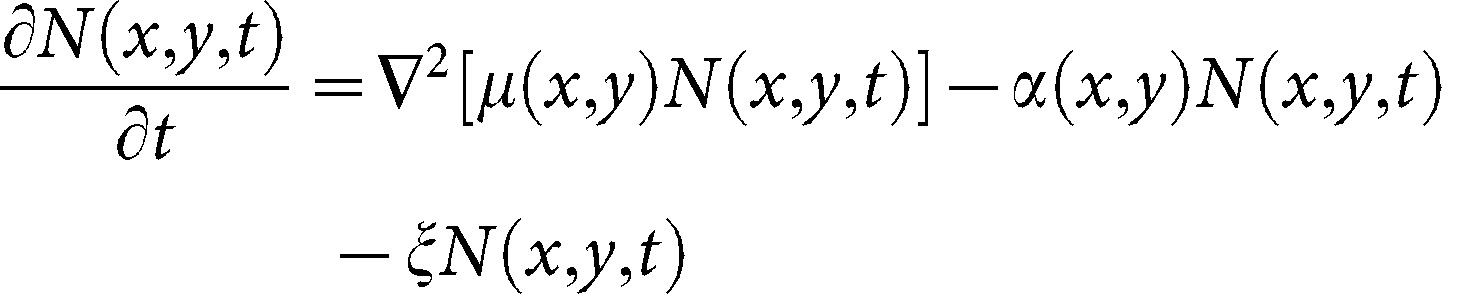
(eqn 1)


**Figure 2:**
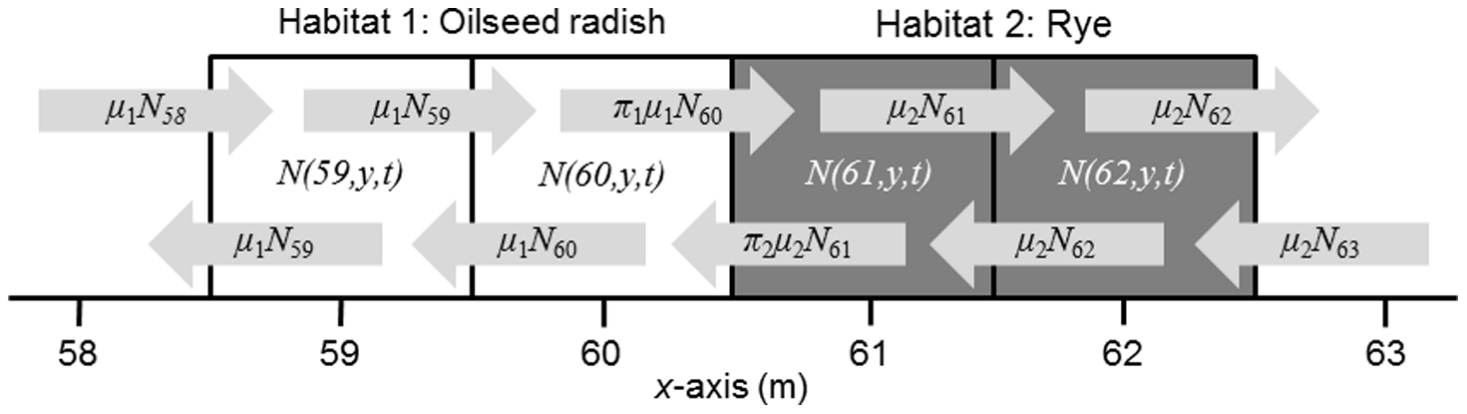
Representation of fluxes in the *x-*direction in the spatial simulation model. *N* (*x,y,t*) is the density of beetles (m^−2^) in a grid cell with coordinates (*x,y*) at time *t. μ*_1_ and *μ_2_* (m^2^ d^−1^) are the motilities of beetles in oilseed radish and rye, respectively. *π*_1_ and *π*_2_ are dimensionless and modify the fluxes of beetles between the two habitats.

In this equation *∇* ^2^ is the Laplace operator that takes the second derivative in the x and y direction, *μ* (*x,y*) is the motility (m^2^ d^−1^), which determines the rate of random movement and can vary spatially according to the local conditions (e.g. the crop). *N* (*x,y,t*) is beetle density (m^−2^) at location (*x,y*) and time (*t*), *α*(*x,y*) is the relative rate of beetle removal by traps, which varies depending upon presence/absence of a trap (hereafter: relative capture rate; d^−1^), and *ξ* is the relative loss rate of marked beetles due to death or mark wear (hereafter: relative loss rate; d^−1^). Just left and right from the interface, *μ* (*x,y*) was multiplied by a flux-modifier (*π*_1_ and *π*_2_, respectively; see below) to simulate interface behaviour (Fig. 2).

Equation 1 was solved numerically using the forward central finite difference method [22] on a lattice of grid cells with mesh size Δ*x* = Δ*y* = 1 m. The change in density of beetles in a grid cell centred on coordinates (*x,y*) during a time step Δ*t* was calculated as:



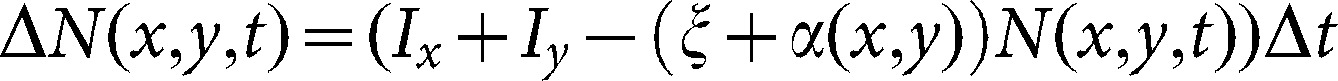
(eqn 2)where *I_x_* and *I_y_* represent the net rate of change of beetle density in a grid cell due to fluxes over the border with adjacent cells in the *x* and *y* directions, respectively. The flux of beetles in the *x*-direction and *y*-direction are shown in equations 3a, b and 4, respectively. In the *x*-direction, different forms of the equation are used at the interface (3b), as compared to elsewhere in the field (3a). Equation 3b includes flux modifiers π_1_ and π_2_ that allow for preferential movement across interfaces.



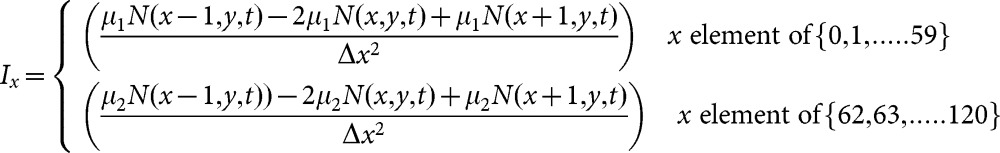
(eqn3a)




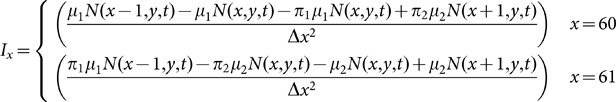
(eqn)

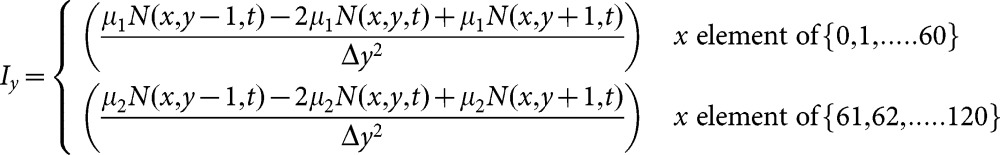
(eqn4)


In the above equations *μ*_1_ is motility in oilseed radish, and *μ*_2_ motility in rye. The dimensionless flux-modifier *π*_1_ affects the flux of beetles from oilseed radish to rye, while *π*_2_ modifies the opposite flux (Fig. 2). The meaning of these flux-modifiers can be understood by considering a beetle that is situated exactly on the interface. Its probability of moving to rye is *π*_1_/(*π*_1_+*π*_2_) while its probability of moving to oilseed radish is *π*_2_/(*π*_1_+*π*_2_). When *π*_1_>*π*_2_ (or *π*_1_<*π*_2_) the direction of movement on the interface is biased towards rye (or oilseed radish). For *π*_1_ = *π*_2_ = 1, movement over the interface is entirely determined by the habitat-specific motilities and densities (see Fig. 2). When *π*_1_ = *π*_2_>1, both fluxes are increased, and when *π*_1_ = *π*_2_<1 both fluxes are decreased, but there is no bias in the behaviour at the interface. High (low) values of the flux modifiers represent an interface that is easy (difficult) to cross. While the relative sizes of the *π*'s determine the bias, their absolute sizes determine the size of the fluxes over the interface, and hence the speed at which the population crosses the interface. Dispersal with each habitat is governed by the motilities μ_1_ and μ_2_.

More beetles are caught in pitfalls if their rate of movement is higher. Relative capture rate *α* (*x,y*) is assumed to be linear related to *μ* according to:



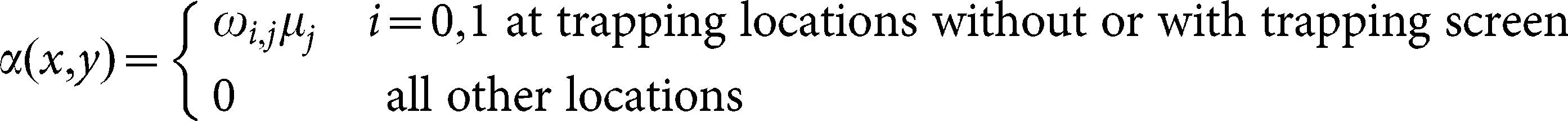
(eqn5)where the constant of proportionality *ω_i,j_* (m^−2^) is the efficiency with which beetles are recaptured at a trapping station with (*i* = 1) or without (*i* = 0) a screen. The index *j* identifies the habitat to which the parameter applies. The parameter fitted is *ω_i,j_* not *α*. Initial model calibrations indicated that there was no support from the data for a habitat specific trapping efficiency. Therefore, we did not include this option in the model selection procedure. The fully parameterized model contained six free parameters, two for motility, one for loss rate, two for trapping efficiency and one for edge behaviour.

The simulated field of grid cells was bordered on all sides by a 1-m wide “slow-release” boundary with a reflective outer edge. This slow-release boundary represents in a crude way the “landscape context” of the experiment. The motility *μ_0_* in this slow-release boundary determines how long beetles are retained in the surrounding landscape before returning to the field. The time step of integration Δ*t* used in solving the model (eqn 2) was one third of the upper value Δ*t*_max_ obtained from the Von Neumann criterion [22]:



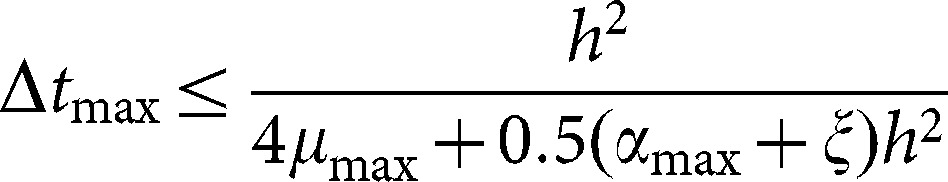
(eqn 6)in which *h* ^2^ = Δ*x*Δ*y*, and *μ*_max_ and *α*_max_ are the maximum values used in model calibration.

#### 2.1.2 Model calibration and model selection

Variants of the model described by equation 2 were calibrated to the data by minimizing the negative log-likelihood: 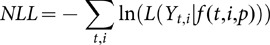
where *L* is the negative binomial likelihood of the data *Y_t,i_*, given model predictions *f* at time *t* and trap location *i*, based on parameter vector *p*. The *NLL* was minimized using a differential evolution algorithm [23], implemented in C++ code that is part of the COMPASS framework [24].

The value of motility in the slow-release boundary *μ*_0_ and the dispersion parameter of the negative binomial error distribution *k* were estimated by calibrating the model (parameterized as model 4 in Table 1) to the data. The calibrated values for *μ*_0_ and *k* were set as constants during the calibration of the other model variants.

**Table 1:** Model selection among variants of the *Fokker-Planck* diffusion model describing beetle dispersal in the field experiment.

			Model parameters					
Model	NLL	ΔAIC	*μ*_1_	*μ*_2_	*ξ*	*ω*_0_	*ω*_1_	*π*_2_
			(m^2^ d^−1^)	(m^2^ d^−1^)	(d^−1^)	(m^−2^)	(m^−2^)	(-)
1	944.0	0.0	215		0.066	0.17	0.09	1.5
2	944.0	2.0	218	212	0.066	0.17	0.09	1.5
3	948.9	7.9	216		0.066	0.15	0.09	
4	949.0	10.0	228	220	0.065	0.15	0.09	
5	959.5	29.1	180		0.071	0.13		1.2
6	960.7	29.6	183		0.071	0.12		
7	959.5	31.1	175	187	0.071	0.13		1.2
8	960.7	31.5	175	194	0.071	0.12		
9	997.3	104.6	217			1.46	0.35	1.8
10	997.3	106.6	219	216		1.47	0.35	1.8
11	1000.9	111.9	165	288		1.27	0.35	
12	1002.1	112.3	214			1.25	0.35	
13	1023.2	154.6	200			0.95		1.5
14	1023.4	154.8	140	316		0.89		
15	1022.7	155.4	159	256		0.94		1.3
16	1025.5	157.1	202			0.86		

A single parameter value is shown when habitats or trap types were not distinguished in a model variant. The value of *π*_1_ is 1 throughout. If no value for *π*_2_ is shown, it was set to 1 and not included in the calibration. The negative log-likelihood (NLL) is a measure of the goodness of fit of the model to the data. ΔAIC is the difference in Akaike's information criterion between a model variant and the model variant with most support of the data (model 1). Models variants of which the ΔAIC is smaller than two are considered equivalent and have equal support from the data.

*μ*_1_: motility in oilseed radish; *μ*_2_: motility in rye; *ξ*: relative loss rate due to removal other than recapture (e.g. mark wear and mortality); *ω*_0_: trap-coefficient for trapping stations without screens; *ω*_1_: trap-coefficient for trapping stations with screens; *π*_1_: multiplication factor of the flux of beetles from oilseed radish to rye; *π*_2_: multiplication factor of the flux of beetles from rye to oilseed radish.

The most complex model for beetle dispersal contained seven parameters (*μ_1_, μ_2_, ξ, ω_0_, ω_1_, π_1_, π_2_*). We fitted 16 alternative models to the data, and used Akaike's information criterion (AIC) to rank these models according to the level of support from the data [20], [21]. AIC was calculated as AIC = 2*NLL*+2*n*, where *NLL* is the negative log likelihood, a measure for goodness of fit, and *n* is the number of parameters. ΔAIC was calculated by comparing a model's AIC to the minimum AIC of the best model. Models that differ less than 2 AIC units have similar support from the data.

### 2.2 Arena experiments

#### 2.2.1 Experimental setting

Movement of individual beetles was video-recorded in autumn 2009 in two arenas of 2×2.5 m with either oilseed radish (*Raphanus sativus* var. Brutus) or winter rye (*Secale cereale* var. Admiraal), in a climate controlled greenhouse. The arenas were filled with 5 cm moist sandy soil collected from the Droevendaal organic experimental farm, on top of 5 cm of potting soil. Similar to agronomic practice the species were sown at 12.5 cm row distance and a sowing density of 30 kg ha^−1^ for oilseed radish and 100 kg ha^−1^ for rye. The species were sown four weeks before the start of recordings.

#### 2.2.2 Beetles

*Pterostichus melanarius* were collected at the end of September 2009 in rye and oilseed radish at the Droevendaal farm using pitfall traps. Beetles were stored in containers (45×30×15 cm) on a substrate of moist potting soil in a climate cabinet with a 12:12 h L:D photoperiod and a 18∶12°C L:D temperature regime, about 200 beetles per container. Over the course of 4 days the photoperiod in the climate cabinet was reversed in two steps of 6 hours. This reversed the activity period of *P. melanarius* and enabled recording during working hours. On 12 October, the temperature regime in the climate cabinet was adjusted to the temperature regime in the greenhouse (20∶15°C L:D). Beetles in the containers were fed frozen fly maggots (*Lucilia caesar*) once every week.

Each week, approximately 100 beetles were collected from the containers for use in recording sessions. These beetles were sexed and transferred to individual plastic cups (Ø 6 cm, 6 cm height) containing some potting soil. Beetles in half of the cups were fed 1–2 maggots twice a week (fed beetles); the other beetles were deprived of food for at least one week before recording (starved beetles).

#### 2.2.3 Video recordings

Video recordings were made from 12 to 20 October 2009 in the dark with a near-infrared radiation source (IR-880/12, 880 nm) (c-tac, Winsen, Germany). Images were captured using a digital camera (Imaging Development Systems GmbH, Obersulm, Germany: uEye UI-1480RE (2560×1920)) from which the infra-red cut filter was removed. To make beetles visible for the camera a small auto-adhesive retro-reflector (35 mm^2^, ∼5 mg; 3M8850, 3M Leiden, The Netherlands) was attached to the elytra [25].

#### 2.2.4 Processing position data

Position data were extracted from the digital images by software written in Matlab R2009a (The MathWorks). Movement tracks were constructed by first excluding all position data that were inside a 10 cm zone from the arena's edge to avoid edge effects caused by wall-following behaviour [26]. Also position changes of less than 0.3 cm were excluded, as these could have been caused by recording error. Next, the position data from the arena's interior were grouped into tracks. A track started when a beetle entered the arena's interior from the edge zone and ended when the beetle returned to the edge zone. A track also ended when the beetle was invisible for more than 20 s. Positions within tracks were aggregated into moves using a data reduction method described by Turchin ([27], p. 132). In this method a chosen distance Δ*z* defines a band width around each move and successive positions within the band are considered to be part of the same move. The first position outside this band defines a new move [27]. Effectively, Δ*z* determines the resolution at which positions are aggregated. For Δ*z* = 0, all original positions are retained, whereas for a large Δ*z* all positions are aggregated into a single move [25]. We used a resolution of Δ*z* = 1.6 cm, which was large enough to prevent autocorrelation in the movement parameters and small enough to retain detail in the movement path.

#### 2.2.5 Analysis of moves

Beetles that made fewer than 50 moves (N = 26) were excluded from the analysis because the calculated movement parameters, especially the mean cosine of turning angles [28] would be inaccurate. For the remaining beetles (48 starved, 49 fed) we calculated average move length *m*_1_ (cm), average squared move length *m*_2_ (cm^2^), average move duration *τ* (s), mean cosine of turning angles (change in direction between subsequent moves in the interval (−*π*, *π*) *ψ* (-), average velocity *v* (cm s^−1^), and motility *μ* (cm^2^ s^−1^). Periods that beetles were invisible or visible but not moving were included in the calculation of the time duration of a move. Motility was calculated for each beetle from the above movement parameters using a formula derived from the Patlak equation Turchin ([27] p. 102):



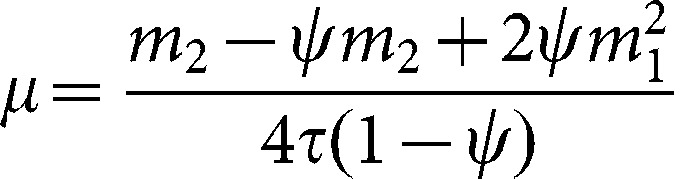
(eqn7)


Motility as a population parameter was calculated by averaging the motilities of individual beetles. The motility estimate that we obtained in the arenas was extrapolated to field scale by assuming that the movement pattern observed in the arenas was representative for the movement pattern during an activity period of 11 h per 24 hours, the time between sunset and sunrise in the Netherlands in September. Accordingly, motility obtained in the arenas (cm^2^ s^−1^) was multiplied by 11×3600×10^−4^ = 3.96 to obtain daily motility (m^2^ day^−1^).

#### 2.2.6 Statistical analysis

A Generalized Linear Mixed Model (GLMM) (GenStat Fourteenth Edition, VSN International Ltd) was used to analyse the effects of feeding level, gender and crop type on the time that beetles spent in the arena's interior and on the movement parameters *m*_1_, *ψ, τ, m*_2_, *v,* and *μ*. Date of recording was included in the model as a random term. To stabilize variance, log- and square root transformations were used and two outliers in the data of move duration were removed (*τ* = 17 s and *τ* = 26 s). The F-statistic was used as a criterion for significance at a 95% confidence level. The total time that beetles spent in the arena's interior was calculated as the sum of path durations (trajectory from edge to edge via the interior). A two-sample Welch's t-test was used to test for a difference between habitats in the mean frequency of beetles moving from the arena's edge zone to the interior, and for a difference between habitats in the mean path duration in the arena's interior. A square root transformation was used to homogenize the variances of the data on path duration.

## Results

### 3.1 Field experiment

Out of the 2030 released beetles, 996 were recaptured over a period of 23 days. Of the beetles released in oilseed radish, 7% were recaptured in rye, and of those released in rye, 12% were recaptured in oilseed radish, indicating greater numbers moving from rye to oilseed radish than vice versa.

Motility of beetles in the slow-release boundary *μ*_0_ and the negative-binomial dispersion parameter *k* were calibrated using variant 4 (Table 1) of the Fokker-Planck model resulting in estimates of *μ*_0_ = 4.1 m^2^ d^−1^ and *k* = 3.1. Preliminary calibrations showed that model credibility depended more on the ratio of the parameters *π*_1_ and *π*_2_ than on their absolute values. For example, optimizing both *π*_1_ and *π*_2_ of model 1 (Table 1) resulted in an AIC that was 1.9 higher than the AIC of the model variant in which only *π*_2_ was optimized, i.e. a small decrease in negative log-likelihood was more than offset by an increase in the penalty for the extra parameter. We concluded that the data did not support the determination of two flux modifiers. We therefore set *π*_1_ to one and calibrated only the value of *π*_2_.

The greatest support by the data was for model 1 (Table 1) with a single motility parameter (*μ* = 215 m^2^ d^−1^; identical for oilseed radish and rye), a relative loss rate of *ξ* = 0.066 d^−1^, trapping efficiencies *ω*_0_ = 0.17 m^−2^ without screens and *ω*_1_ = 0.09 with screens, and a flux modifier from rye to oilseed radish *π*_2_ = 1.5, indicating preference for oilseed radish.

**Figure 3:**
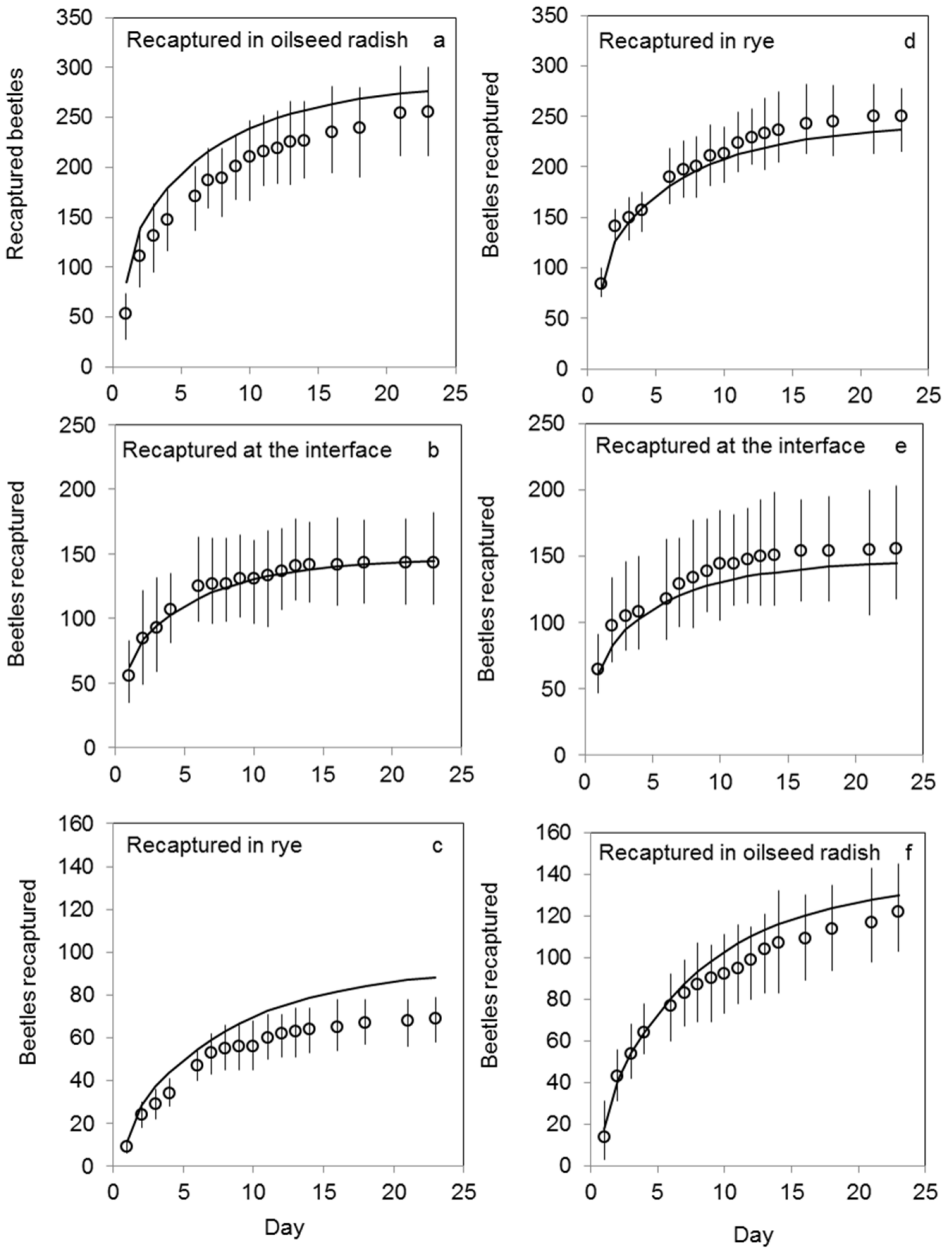
Observed (o) and predicted (line) cumulative number of recaptured *P. melanarius* as a function of time. Predicted values were simulated with the calibrated model that had most support from the data (model 1, Table 1). Panels on the left (a–c) are for beetles released in oilseed radish, on the right (d–f) for beetles released in rye. Error-bars show the 95% confidence interval of the observations.

Correspondence between model and data was evaluated by first comparing the simulated and observed trap catches, summed per crop (and the interface), and cumulated over time. The comparison between simulated and observed for the beetles released in radish showed some overestimation by the model of trap catches in radish (Fig. 3a), and good correspondence for pitfalls at the interface (Fig. 3b) and in rye (Fig. 3c). For beetles released in rye the predictions were well within the 95% confidence interval of the data (Fig. 3d–f).

**Figure 4:**
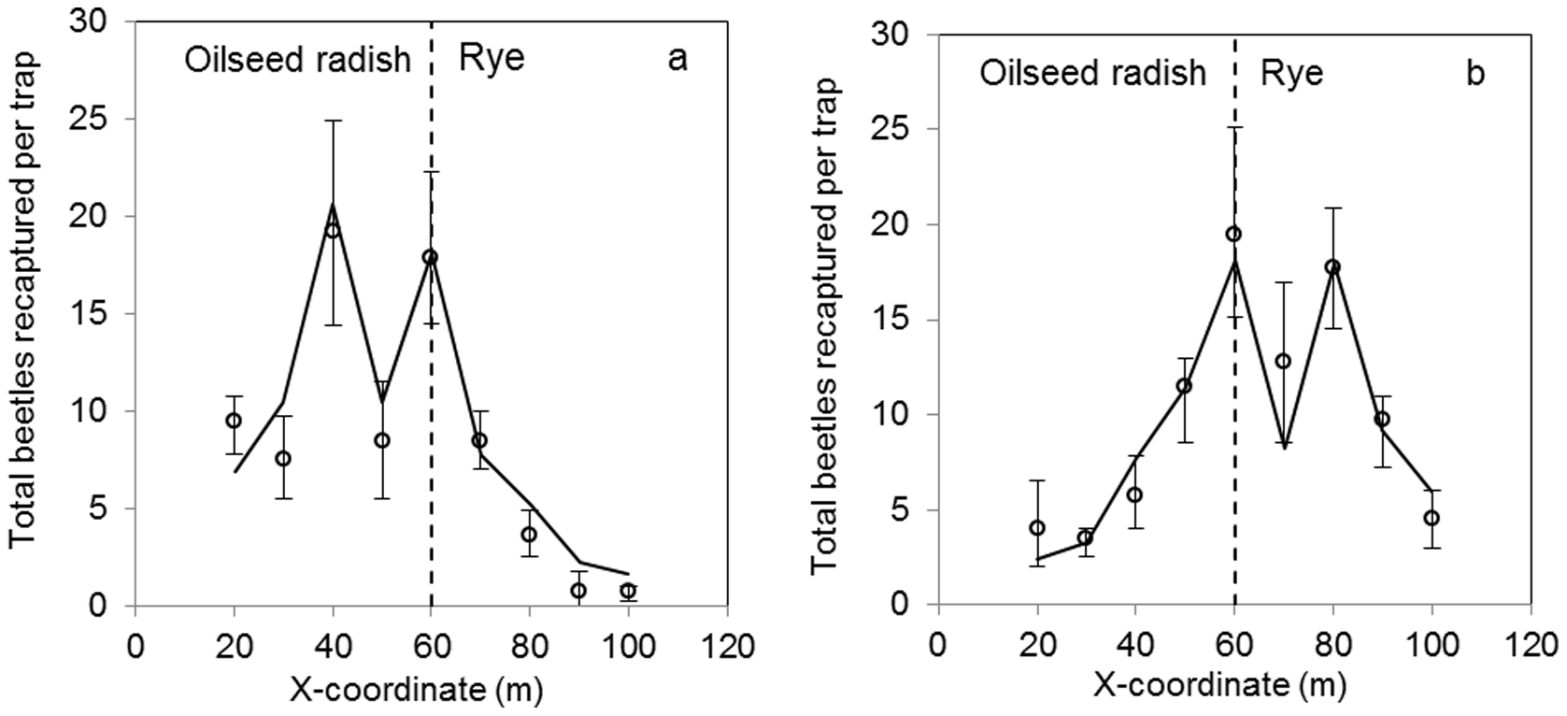
Distribution of observed (o) and predicted (line) number of *P. melanarius* recaptured along the length of the experimental field, cumulated over time and over the width of the field for (a) beetles released in oilseed radish at 50 m and (b) beetles released in rye at 70 m. Predicted values are simulated with the model that had most support from the data (model 1, Table 1). The error-bars show the 95% CI of the observations. Averaging of densities at a given *x* was done over all traps at that *x*-value. At x = 50 and 70 m, there were no traps close to the release points, causing a drop in the catch, both in the data and the fitted model. Cf. Fig. 1 for trap locations.

**Figure 5:**
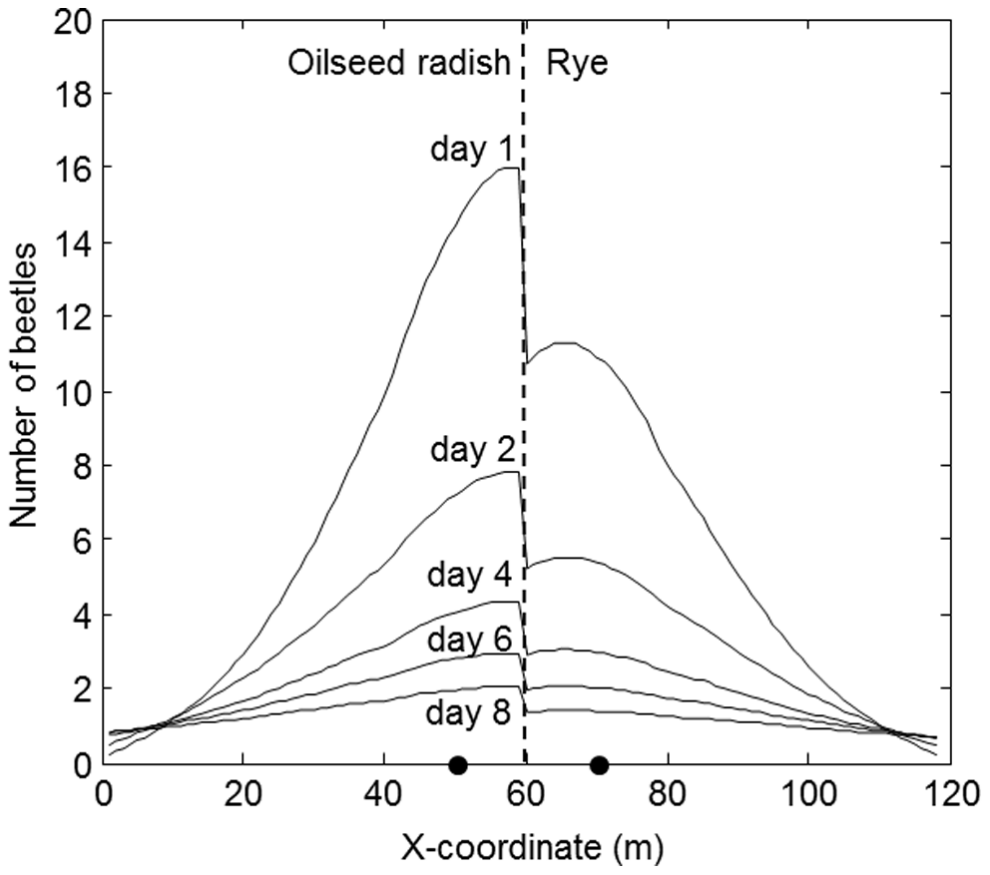
Distribution of *P. melanarius* along the length of the field over time simulated with the model that had most support from the data (model 1, Table 1). The Y-axis represents the total number of beetles over the width of the field. On day 0, 1015 beetles were released at each black dot. Number of beetles in the slow-release margins that surround the field are not shown.

Comparison of the time-integrated simulated and observed catches in space (*x*-direction) is shown as marginal totals (integrated over the y-direction) in Fig. 4. There was good correspondence between simulations and observations, both for the beetles released in radish (Fig. 4a) and those released in rye (Fig. 4b). The dent in the total number of beetles recaptured per trap at 50 m and 70 m (the *x*-coordinates of the release) is due to absence of traps close to the release points. In other words: at these *x*-locations the traps were only placed at the “other” side of the field, i.e. at a subset of all different values of *y* (see Fig. 1). Simulations show discontinuities in density at the habitat interface as a consequence of the greater preference of beetles for radish as compared to rye. The ratio of the number of beetles just left (oilseed radish) to just right (rye) of the interface was constant at 1.5 (Fig. 5).

**Figure 6:**
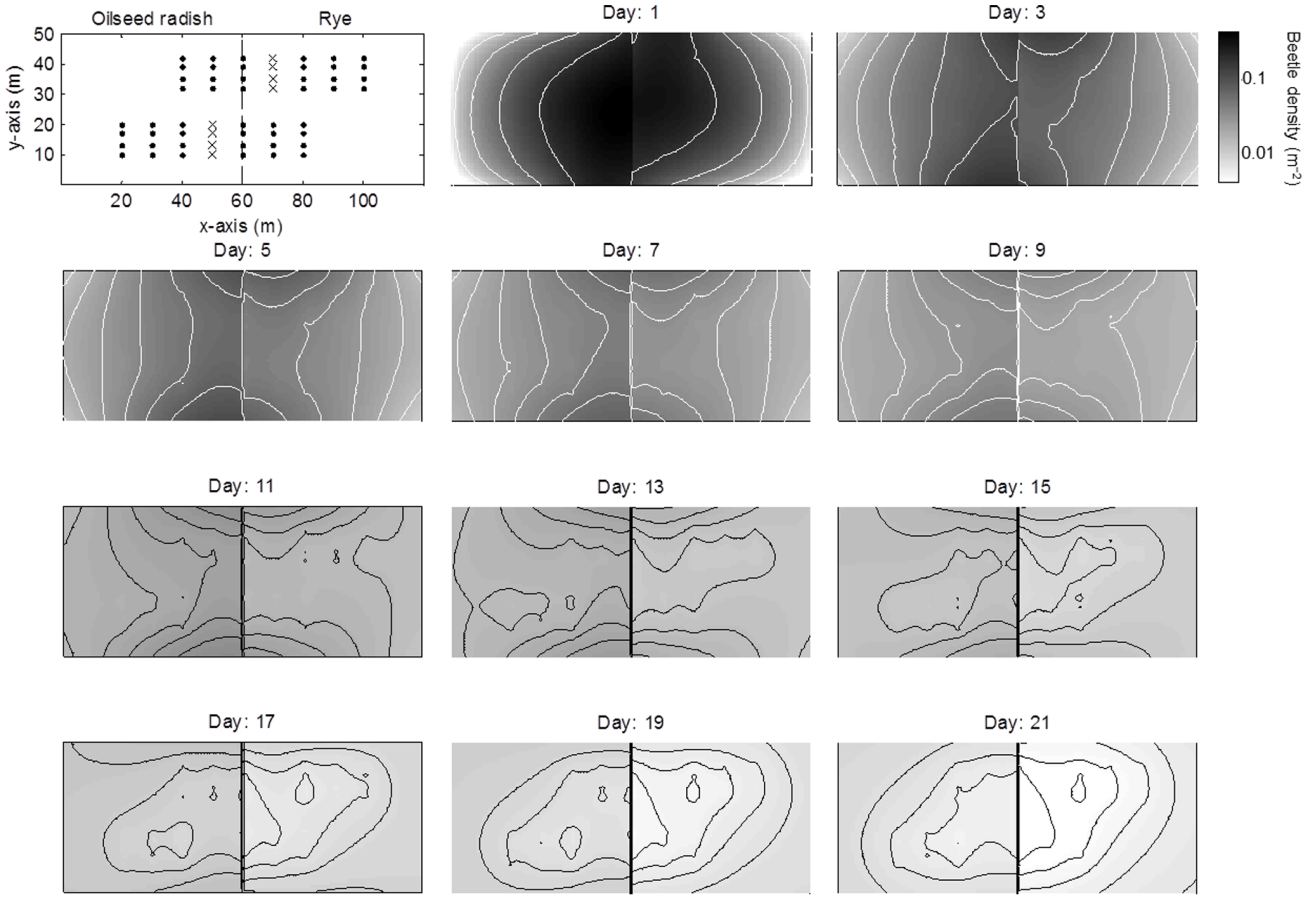
Time evolution of the distribution of *Pterostichus melanarius* in two adjacent crops simulated with a Fokker-Planck diffusion model (model 1, Table 1) that had a common value of motility for both crops (215 m^2^ d^−1^) and preferential movement at the interface equivalent to a beetle on the interface moving to oilseed radish with a probability of 0.60 and moving to rye with a probability of 0.40. Beetle densities varied between 0.42 m^−2^ on day 1 to 0.008 m^−2^ on day 21. Beetles densities in the slow-release margins that surrounded the experimental field were omitted. Crosses and dots in the upper left panel mark the locations at which beetles in the model were released and recaptured, respectively.

**Figure 7:**
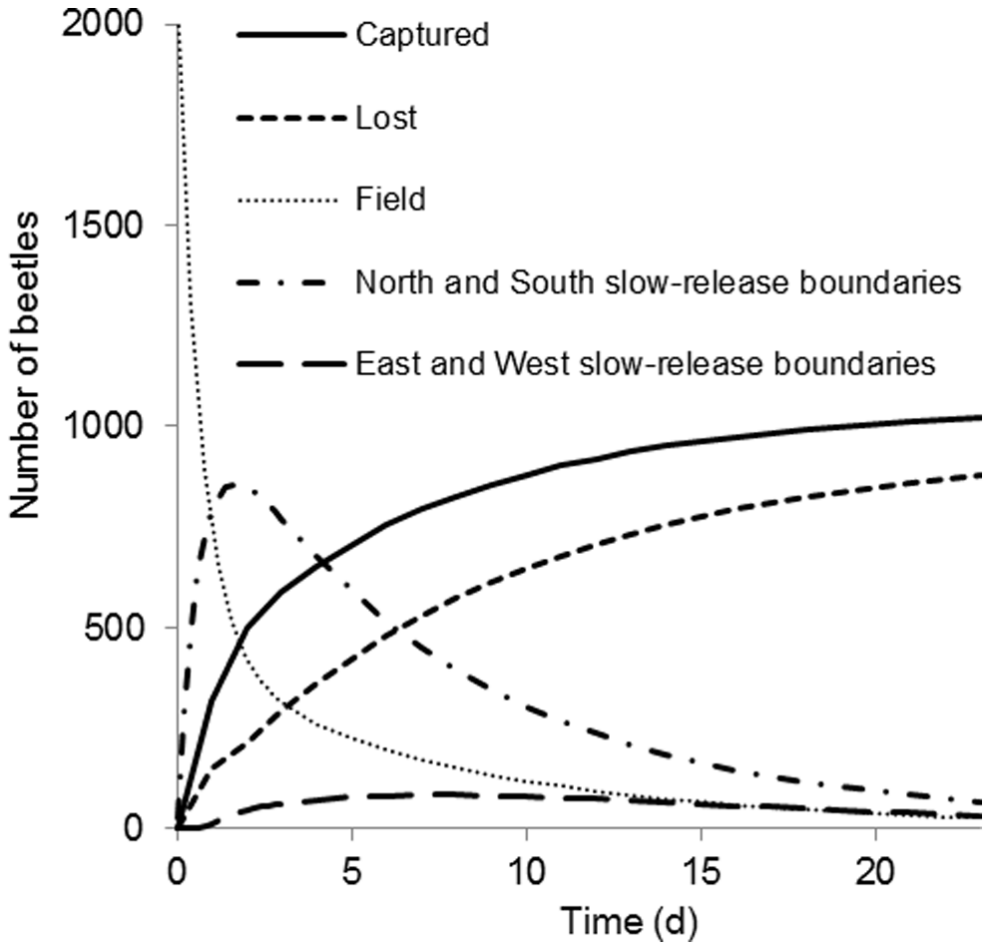
Simulated change in time of the number of marked beetles in the field and the slow-release boundaries, and the change in number of beetles that were either captured or lost due to mark wear or death. Simulations were made with the model that had most support from the data (model 1, Table 1). At Time  = 0, a total of 2030 virtual beetles were released.

The total number of beetles in the field declined through time as a result of mortality, mark wear, pitfall catch and movement across the edge of the field into the slow release boundary. (Fig. 6). A substantial proportion of dispersing beetles reached the field edges in the simulations. On the first day, 757 of the released beetles (37%) were still in the experimental field (Fig. 7). The remaining beetles had moved into either the north or south boundaries (39%), were recaptured (16%), were lost due to mark wear or mortality (7%) or had moved into the east or west slow-release boundaries (1%) (Fig. 7). By the end of the experiment most beetles had been recaptured or lost due to e.g. mark wear or mortality, and only a small fraction was still in the field or in the slow-release boundaries.

### 3.2 Arena experiment

**Figure 8:**
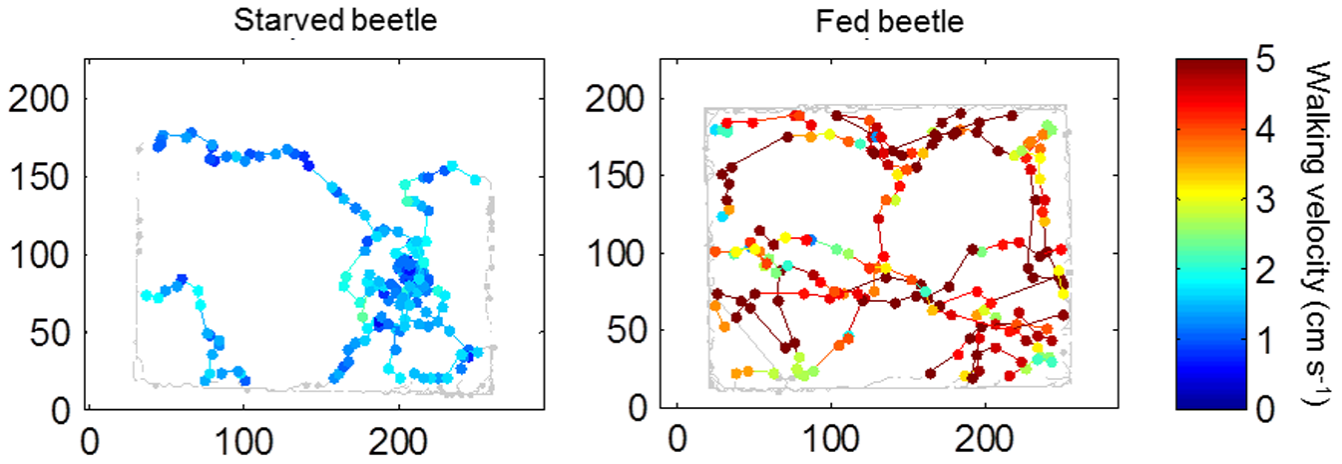
Examples of movement tracks for a starved *Pterostichus melanarius* beetle with low motility (7 cm^2^ s^−1^; left) and for a fed beetle with a high motility (88 cm^2^ s^−1^; right). The total time spent in the arena's interior was 24.8 min for the starved and 11.7 min for the fed beetle. Moves in the 10 cm edge zone are represented by thin grey lines.

GLMM analysis demonstrated a significant effect (*p*<0.05) of feeding level on all movement parameters except angular dispersion, and no significant effects of gender or crop species (Table 2). Motility, move length, squared move length, move duration and speed were significantly higher for fed than for starved beetles (Fig. 8, Table 2). Average daily motility of beetles in the arenas ranged between 147 and 207 m^2^ d^−1^ (Table 2), which was similar to the motility of beetles in the field experiment (215 m^2^ d^−1^).

**Table 2:** Average movement parameters (± se) of *P. melanarius* in an arena experiment with hunger level, gender and crop species as factors.

	Starved (N = 48)	Fed (N = 49)	d.d.f.	*F* *	*p* two- sided
Time in arena's interior (min)	15.8±1.2	11.6±0.8	91.7	8.6	**0.004**
Move length *m*_1_ (cm)	10.9±0.4	11.7±0.4	90.7	7.3	**0.008**
Angular dispersion *ψ* (-)	0.707±0.011	0.700±0.013	92.4	0.01	0.940
Move duration *τ* (s)	7.3±0.5	5.6±0.2	89.5	9.8	**0.002**
Squared move length *m*_2_ (cm^2^)	165.0±11.9	188.3±12.3	90.4	7.0	**0.01**
Speed *v* (cm s^−1^)	1.7±0.1	2.2±0.1	92.9	12.6	**<0.001**
Motility *μ* (cm^2^ s^−1^)	38.3±5.1	50.3±6.8	91.3	8.8	**0.004**
Daily motility *μ* (m^2^ d^−1^)	151.7±20.2	199.2±26.9			
	Female (N = 50)	Male (N = 47)	d.d.f.	*F* *	*p* two- sided
Time in arena's interior (min)	14.0±1.0	13.4±1.2	91.8	0.7	0.409
Move length *m*_1_ (cm)	11.2±0.3	11.4±0.5	90.4	0.2	0.704
Angular dispersion *ψ* (-)	0.701±0.009	0.706±0.014	92.2	0.2	0.650
Move duration *τ* (s)	6.2±0.2	6.6±0.6	88.2	1.3	0.260
Squared move length *m*_2_ (cm^2^)	172.2±8.2	181.8±15.5	90.1	0.2	0.702
Speed *v* (cm s^−1^)	1.9±0.1	2.1±0.1	92.8	2.1	0.154
Motility *μ* (cm^2^ s^−1^)	37.1±2.9	52.2±8.2	91.0	0.2	0.696
Daily motility *μ* (m^2^ d^−1^)	146.9±11.5	206.7±32.5			
	Oilseed radish (N = 53)	Rye (N = 44)		*F* *	*p* two- sided
Time in arena's interior (min)	15.6±1.1	11.4±1.0	87.3	12.2	**<0.001**
Move length *m*_1_ (cm)	11.5±0.3	11.1±0.4	85.7	0.4	0.521
Angular dispersion *ψ* (-)	0.704±0.011	0.702±0.014	86.3	0	0.906
Move duration *τ* (s)	6.6±0.4	6.3±0.4	85.7	0.5	0.483
Squared move length *m*_2_ (cm^2^)	180.0±9.5	173.0±15.2	85.7	0.9	0.352
Speed *v* (cm s^−1^)	2.0±0.1	2.0±0.1	86.4	0.1	0.737
Motility *μ* (cm^2^ s^−1^)	41.5±4.1	47.9±8.1	85.9	0	0.952
Daily motility *μ* (m^2^ d^−1^)	164.3±16.2	189.7±32.1			

Number of observations per factor are indicated in the column headings. Beetles were recorded for 50 min in arenas of 5 m^2^. *P*-values in bold indicate significant differences (*p*<0.05) between treatment groups.

*F statistic, n.d.f. = 1 for all tests.

During the 50 min recording period beetles moved between the edge-zone and the arena's interior (Fig. 8). GLMM analysis demonstrated a significant effect of feeding level and crop type, but not of gender on the total time beetles spent in the arena's interior (Table 2). Beetles spent more time in the interior of oilseed radish than of rye because beetles moved significantly more often from the edge into the interior of oilseed radish (mean ± se: 17.0±1.1 times) than of rye (13.5±1.0 times) (t-test: t = 2.3, d.f. = 95, p = 0.024). Furthermore, the average path duration in the interior was also greater in oilseed radish than in rye (oilseed radish: mean ± se: 1.9±0.9 min; rye: 1.1±0.1 min; t-test: 7.1, d.f. = 93.2, p<0.001). These results indicate a preference of beetles for oilseed radish over rye and an inclination of beetles to stay in oilseed radish. Between fed and starved beetles there was no significant difference in the frequency of moves from the edge into the interior (mean ± se: 15.0±1.1 times for starved beetles and 15.9±1.1 for fed beetles; t-test: t = 0.6, d.f. = 95, p = 0.554).

The mean path duration of starved beetles in the arena's interior was significantly greater than the mean path duration of fed beetles (mean ± se: 2.2±1.0 min for starved beetles; 0.8±0.07 min for fed beetles; t-test: t = 2.1, d.f. = 95, p = 0.039).

## Discussion

Both the field and arena results provide evidence for preference behaviour of *P. melanarius* at the habitat interface. In the field significantly more beetles moved from rye to oilseed radish than in the opposite direction, which was best described by a diffusion model that contained preference behaviour at the interface and used the same motility in both habitats. Also in the arena motility of beetles was not different between the crop species and beetles entered more frequently into the vegetated zone and were more reluctant to leave this zone in oilseed radish than in rye. The interface between oilseed-radish and rye thus significantly influenced behaviour of *P. melanarius* in the field.

The preference of beetles to move to oilseed radish may be due to two mechanisms, attraction towards oilseed radish, a greater tendency of beetles to stay in this crop, or both. The arena observations provided evidence for both of these mechanisms. A greater preference for oilseed radish compared to rye may be caused by a response of *P. melanarius* to differences in plant odours [29] or differences in micro-climate [30], [31]. While plant odours may attract insects over larger distances, a change in movement behaviour in response to micro-climate operates at a local scale.

Our approach to boundary behaviour with flux modifiers is slightly different to the approach described in Ovaskainen [7]. The difference is that with the flux modifiers we identify motility and preference at the boundary separately, while with the habitat selection approach in Ovaskainen [7] the combined effect of both parameters for the density at the interface is estimated [9]. When motility is equal on both sides of an interface our flux modifiers are equivalent to the habitat selection parameters in Ovaskainen [7]. In that case the change in density at the interface is given by the ratio of the two flux modifiers π_1_: π_2_ or by the ratio of the two habitat selection parameters k_1_: k_2_ in Ovaskainen [7] or by the ratio (1+z): (1–z), where −1≤z≤1 measures the preference of an individual for either side of the interface in Ovaskainen and Cornell [9]. The boundary condition in our study is thus the same as in Ovaskainen [7] and Ovaskainen and Cornell [9] and depends on a single parameter: the ratio of two boundary multipliers, i.e. one preference parameter.

For the outer boundaries of the field we implemented a ‘slow-release boundary’ for which a specific motility parameter was calibrated. Simulation results showed that due to their high motility, many beetles reached the north and south boundaries of the field, which first acted as sinks and later as sources of beetles (Fig. 7), just as could happen in a real landscape. The north and south boundaries consisted of six-meter wide grass strips which are known to slow down movement of carabid beetles [32], [33], [34], [35]. The predicted accumulation of beetles in the margins by the model is thus a realistic reflection of an experimental landscape setting.

In the arenas we observed a large variation in movement behaviour between individuals. Some beetles made straight lines from one side of the arena to the other, while others made very tortuous movements. The behaviour of individuals was linked to population re-redistribution using motility as an intermediate variable. The variation in behaviour between individuals played herein an important role. When the variation in movement behaviour between individuals would be ignored, e.g. by pooling movement data of all individuals, linking movement to population re-distribution may underestimate the true rate of population spread due to the non-linear (convex) relation between movement parameters (e.g. step lengths and turning angles) and motility (i.e. the rate of population spread) in equation 7. The error made by calculating motility (eqn. 7) from the mean of the movement data of all individuals rather than – as it should be done – by calculating motility of the movement data of each individual and then calculate the mean output, is known as “Jensen's inequality” [36]. The error caused by pooling movement data can be large. For instance, in our experiments motility of starved beetles was 151.7 m^2^ d^−1^, but would have been 106.2 m^2^ d^−1^ if motility had been calculated from movement data pooled over individual beetles. Neglecting variation in individual variation in behaviour may thus underestimate the true rate of population spread. This is relevant for ecologists who use individual based simulations of animal movement to make predictions on population level processes.

In this study we combined observations on behaviour of individual beetles in arenas and on behaviour of a population of released beetles. The link between these different approaches shows in an elegant way how individual preferences are reflected in the population redistribution by diffusion: the motility of beetles in the arenas scaled-up to daily motility corresponded well with the motility of beetles estimated from our field data. In the arenas motility was calculated for individual beetles based on 50 min observation time. In the field study a single value for motility was estimated using inverse modelling with a Fokker-Planck diffusion model, describing the average rate of population spread over a period of 23 days. The close correspondence in motility between the field and arena indicates that the behaviour of beetles in the arenas gave a good representation of behaviour of beetles in the field.

In conclusion, our study provides details on the behavioural processes that lead to pattern formation of arthropods in agricultural land by scaling up individual movement behaviour to population spread. We show that the interface between crop habitats affect the dispersal and distribution of carabids in adjacent crops. Our dual approach provides a framework for evaluation of movement within and across habitats in more complex agricultural landscapes.
